# Assessing Time–Frequency Analysis Methods for Non-Stationary EMG Bursts: Application to an Animal Model of Parkinson’s Disease

**DOI:** 10.3390/s26051688

**Published:** 2026-03-07

**Authors:** Fernando Daniel Farfán, Ana Lía Albarracín, Leonardo Ariel Cano, Eduardo Fernández

**Affiliations:** 1 Neuroscience and Applied Technologies Laboratory (LINTEC), Bioengineering Department, Faculty of Exact Sciences and Technology (FACET), National University of Tucuman, Superior Institute of Biological Research (INSIBIO), National Scientific and Technical Research Council (CONICET), Av. Independencia 1800, San Miguel de Tucuman 4000, Argentina; lcano@herrera.unt.edu.ar; 2 Institute of Bioengineering, Universidad Miguel Hernández of Elche, 03202 Elche, Spain; e.fernandez@umh.es; 3 Research Networking Center in Bioengineering, Biomaterials and Nanomedicine (CIBER-BBN), 28029 Madrid, Spain

**Keywords:** electromyography, non-stationarity signal processing, time–frequency biomarkers

## Abstract

Time–frequency (TF) characterization of electromyographic (EMG) bursts is essential for accurately assessing muscle function, particularly when the signals exhibit a high degree of nonstationarity. In this exploratory study, we investigated the temporal dynamics of the spectral components associated with short-latency EMG bursts using several TF analysis techniques. Specifically, we compared the performance and interpretability of spectrograms obtained via the short-time Fourier transform (STFT), the continuous wavelet transform (CWT), and noise-assisted multivariate empirical mode decomposition (NA-MEMD), applied to EMG signals recorded from the biceps femoris muscle of freely moving rats in an animal model of Parkinson’s disease, acquired using chronically implanted bipolar electrodes during treadmill locomotion. For each method, we evaluated its effectiveness in capturing transient variations in frequency content, the stability of extracted features across bursts, and the extent to which these features reflect physiologically meaningful aspects of muscle activation. The results show that TF approaches reveal complementary information about burst structure; NA-MEMD provides greater adaptability to nonlinear and nonstationary components, whereas STFT- and CWT-based representations offer more controlled and comparable analyses. Overall, these findings highlight the value of TF analysis as a methodological tool for evaluating muscle function and provide a solid foundation for selecting analytical strategies in studies where EMG bursts exhibit complex and highly variable spectral profiles.

## 1. Introduction

Electromyography (EMG) is a well-established tool for characterizing muscle function due to its high temporal resolution in capturing activation patterns during motor tasks [1]. The temporal evolution of contractile dynamics in both isometric and isotonic contractions reflects the collective activity of motor units [2], which exhibit inherently nonstationary behavior [3].

During isometric contractions, EMG signals are typically analyzed over short time intervals (0.25–2 s) in which quasi-stationary conditions can be assumed [4,5], allowing the extraction of classical metrics such as mean amplitude or mean/median frequency [2]. However, isotonic or dynamic contractions involve muscle fiber displacement, variations in active motor units, and changes in muscle length, generating transient nonstationary intervals with rapid amplitude and spectral fluctuations [6]. Although isometric protocols facilitate controlled comparisons across subjects and sessions, they do not fully capture the complexity of motor-unit recruitment during functional movements and may mask early motor deficits. In neurodegenerative conditions such as Parkinson’s disease, alterations in muscle activation often emerge preferentially during dynamic rather than static tasks [7,8].

Dynamic contractions are characterized by transient and rhythmic motor-unit recruitment, giving rise to EMG bursts that dominate functional and time-demanding movements [9]. In rodents, chronic EMG recordings during free locomotion provide a sensitive approach for assessing contractile dynamics, often revealing functional alterations not detected by behavioral tests [9,10,11,12]. The analysis of these bursts enables the inference of recruitment efficiency, neuromuscular coordination, and pathological alterations [11,13]. Classical spectral parameters, including mean and median frequency, have been proposed as biomarkers of muscle dysfunction in 6-OHDA-lesioned rats, although their validity depends on the partial fulfillment of stationarity assumptions [9,10].

The pronounced nonstationarity of EMG bursts poses significant analytical challenges, often requiring preliminary stationarity assessment before applying conventional feature extraction methods. In a previous study, we introduced a methodological framework based on mean, variance, and autocovariance stationarity tests (MVA-test) to characterize temporal changes in EMG dynamics [10]. In Parkinsonian animal models, this approach revealed a progressive increase in nonstationary components following dopaminergic lesion, underscoring the need for analytical strategies that explicitly account for time-varying spectral structure prior to biomarker extraction.

While preliminary stationarity assessment provides useful information, it does not fully capture the joint temporal and spectral evolution of EMG bursts. Despite these advances, there remains a need for analytical frameworks capable of explicitly integrating the time–frequency dimension, enabling the joint characterization of the temporal and spectral evolution of EMG bursts. Beyond the study of neuromuscular alterations in disease models, advanced time–frequency analysis of EMG signals has also gained increasing relevance in applied technological domains [14]. In rehabilitation engineering, myoelectric prosthesis control, assistive device regulation, and human–machine interface systems, robust feature extraction from highly nonstationary EMG signals is essential for accurate intention decoding and adaptive control. In these contexts, the ability to capture transient spectral dynamics and multiscale temporal patterns may enhance classification performance, improve controller responsiveness, and contribute to more natural and stable device operation [15]. Therefore, methodological advances in time–frequency EMG characterization may have translational implications extending beyond experimental neuroscience toward neuroengineering and rehabilitation technologies [16].

Time–frequency (TF) approaches are particularly suited to this goal, as they provide dynamic representations of transient and highly nonstationary signals. Among these techniques, consolidated methods such as the spectrogram based on the short-time Fourier transform (STFT) [17] and the continuous wavelet transform (CWT) [18] offer complementary time and frequency resolutions. More recently, advanced approaches such as noise-assisted multivariate empirical mode decomposition (NA-MEMD) [19], a multichannel extension of the empirical mode decomposition method, have emerged with the aim of decomposing signals into adaptive intrinsic mode functions (IMFs).

The utility of NA-MEMD in EMG analysis lies in its ability to separate oscillatory components embedded in noisy and strongly nonstationary signals while preserving multivariate interdependencies across channels [20]. By subsequently applying the Hilbert transform to each IMF, it is possible to estimate instantaneous frequency, energy, and other dynamic attributes that are particularly informative in muscle signals, where multiple physiological generators coexist, such as motor units with different firing rates and recruitment strategies [21].

Although the direct physiological interpretation of each IMF remains an open research question and an unequivocal biological meaning cannot yet be assigned [6], their analysis is not devoid of conceptual grounding. Given that surface EMG represents a superposition of the activity of numerous motor units, each with partially independent activation patterns, it is reasonable to hypothesize that certain modes may reflect underlying physiological components, such as frequency groupings associated with neuronal synchrony, modulation of central drive, or changes in motor-unit recruitment and firing rate [22]. Therefore, the systematic exploration of these adaptive modes represents a promising methodological avenue for advancing the time–frequency characterization of EMG bursts and deepening our understanding of the muscle mechanisms that generate them.

In this study, we propose a structured comparative evaluation of three time–frequency methodologies applied to EMG bursts exhibiting high nonstationary content in a 6-OHDA-induced Parkinson’s disease model. Rather than assuming the superiority of a particular technique, the objective of this study is to determine under which analytical conditions each approach provides the most informative and physiologically meaningful representation of transient EMG dynamics. To this end, the comparison is structured around explicit methodological criteria, including sensitivity to rapid temporal–spectral transitions within bursts, stability of extracted features across repetitions, and degree of physiological interpretability. Through this framework, we aim to establish objective guidelines to support the selection of appropriate time–frequency tools for the analysis of highly nonstationary EMG signals, contributing to a more robust characterization of muscle function in disease models and, potentially, in translational neuromuscular assessment and rehabilitation contexts.

The main contributions of this work are threefold. First, we provide a systematic comparative assessment of linear time–frequency representations (STFT-based spectrogram and CWT) and an adaptive decomposition approach (NA-MEMD coupled with Hilbert transform analysis) for the characterization of highly nonstationary EMG bursts. Second, we demonstrate that these methodologies offer complementary insights, with linear approaches revealing global spectral reorganization patterns and NA-MEMD-based analysis uncovering local and transient dynamics that may remain concealed in traditional representations. Third, we show that dopaminergic degeneration induces phase-dependent and scale-specific alterations in EMG frequency modulation, supporting the integration of multiscale time–frequency metrics as a promising strategy for identifying functional biomarkers of neuromuscular reorganization.

## 2. Materials and Methods

This section describes the experimental model, EMG acquisition procedures, gait phase characterization, and the time–frequency analysis methods employed to study nonstationary electromyographic bursts.

### 2.1. Biceps Femoris Electromyographic Activity (BF EMG Activity)

The EMG burst signals analyzed in this study belong to an experimental protocol aimed at evaluating muscle function over time in rats subjected to a neurotoxic model of Parkinson’s disease (PD) [9]. Adult male Wistar rats (N = 24), weighing between 250 and 300 g at the time of surgery, were used. Five animals served as controls, while nineteen rats underwent a unilateral neurotoxic PD model induced by 6-hydroxydopamine (6-OHDA). The toxin was stereotaxically injected into the substantia nigra pars compacta of the right hemisphere, producing contralateral motor impairment affecting the left hindlimb, from which all electromyographic recordings were obtained.

Lesion effectiveness was confirmed three weeks after surgery using the apomorphine-induced rotation test (0.25 mg/kg, subcutaneous), a well-established behavioral assay whose rotational score correlates with the extent of dopaminergic neuron loss. Only animals exhibiting clear contralateral rotational behavior and exceeding 100 turns within 30 min were included in the study, ensuring a homogeneous level of lesion severity across subjects. After confirmation of the lesion, animals were implanted with chronic EMG electrodes and allowed to recover before data acquisition. Recordings were conducted at defined post-lesion stages (Weeks 3 to 6), following a prior habituation and treadmill training period to ensure stable locomotor performance during EMG acquisition.

A previously constructed circular treadmill was adapted to facilitate electrophysiological recordings in freely moving rodents [23]. An array of EMG electrodes was designed to record the activity of small hindlimb muscles. The electrodes were made of stainless steel with a contact surface area of approximately 2.6 mm^2^. Two electrodes were used in a bipolar configuration for EMG recordings, while a separate wire served as the ground electrode. Chronic electrode implantation was performed under general anesthesia using ketamine and xylazine. After a recovery period, the rats were placed on the treadmill for data collection. Each recording session lasted at least 2 min to ensure an adequate number of muscle contractions for precise analysis.

EMG signals were acquired using a commercial acquisition system (Biopac Systems Inc., Goleta, CA, USA) with a sampling frequency of 2000 Hz. The recorded signals were amplified with a gain of 60 dB and analogically band-pass filtered between 30 and 500 Hz. In addition, a 50 Hz notch filter was applied to attenuate power-line interference. This configuration was selected to ensure an adequate signal-to-noise ratio and to preserve the spectral content relevant for surface EMG analysis during dynamic locomotor tasks.

The accuracy of electrode implantation sites was verified at the end of the recordings following euthanasia of each animal. In all animals, electrodes were confirmed to be positioned exactly at the designated implantation sites as previously described [9,23].

### 2.2. Gait Phases and Evoked EMG Activity

**Figure 1:**
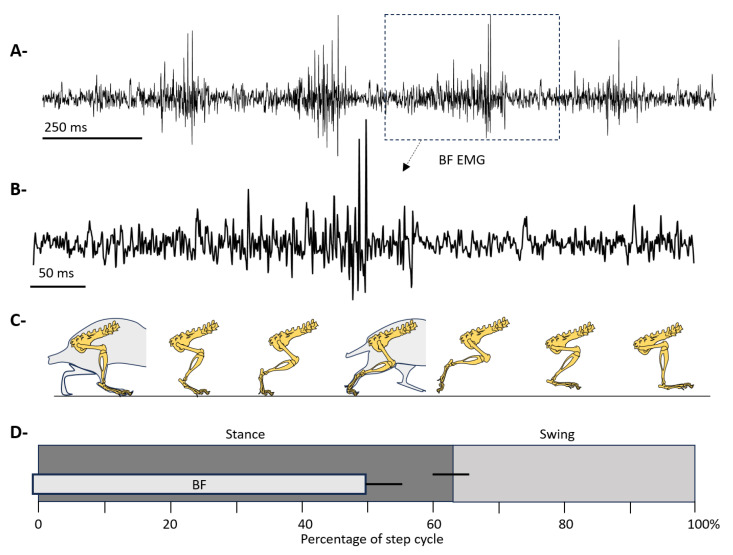
Gait cycle phases and biceps femoris electromyographic activity in the rat. (**A**) Raw BF EMG signal recorded during locomotion, corresponding to four consecutive step cycles. The dashed box highlights a representative segment of the signal that is expanded in panel (**B**). (**B**) Expanded view of the BF EMG signal corresponding to a complete gait cycle, illustrating the temporal morphology of the phasic muscle activity during locomotion. (**C**) Representative sequence of hindlimb positions during a complete gait cycle in the rat, showing the transition between stance and swing phases. (**D**) Schematic representation of the gait cycle expressed as a percentage of the full cycle (0–100%). Gray bars indicate stance and swing phases. The central bar represents the average activation interval of the BF EMG signal, with black lines indicating the observed temporal variability (modified from [25]).

Figure 1 presents a descriptive schematic illustrating the relationship between gait cycle phases and biceps femoris (BF) activation in Wistar rats during spontaneous locomotion. The kinematic sequence of the hindlimb, obtained from video recordings, allows identification of the stance and swing phases within the gait cycle (Figure 1A) [24]. This sequence qualitatively describes the postural progression of the hindlimb throughout the gait cycle.

Additionally, a temporal diagram of the gait cycle, expressed as a percentage of a complete step, highlights the stance and swing phases. Superimposed on this diagram is the average EMG activation window of the BF, indicating the interval in which the main muscle activity burst was observed during locomotion [25]. This information situates the functional role of the BF relative to step biomechanics, particularly its involvement in the transition from late stance to the initiation of the swing phase (Figure 1B). Finally, a representative raw BF EMG signal is shown for a typical burst recorded in a control animal during an experimental session. This trace is provided for illustrative purposes only, highlighting the temporal morphology of BF EMG activity.

### 2.3. Time–Frequency Methods

This subsection describes the three time–frequency methodologies evaluated in this study, including linear representations and adaptive decomposition approaches, together with the corresponding feature extraction and statistical analysis procedures.

#### 2.3.1. Spectrogram via Short-Time Fourier Transform

The spectrogram is a widely used time–frequency representation for the analysis of nonstationary signals and is obtained from the short-time Fourier transform (STFT). The STFT enables the study of the spectral evolution of a signal *x*(*t*) over time by first applying a temporal window *ω*(*t*−*τ*) and then computing the Fourier transform of the portion of the signal weighted by that window. One commonly used time–frequency representation is the STFT [26], defined as:

(1) $$\begin{array}{c} {STFT}_{x}\left(\tau ,f\right)=\int_{-\infty }^{\infty }x\left(t\right)\times \omega \left(t-\tau \right)\times e^{-j\times 2\pi \times f\times t}dt\\ =\int_{-\infty }^{\infty }x\left(t\right)\times \omega_{t,f}^{*}\left(t\right)dt \end{array}$$

where $\omega_{t,f}^{*}\left(t\right)=\omega \left(t-\tau \right)\times e^{-j\times 2\pi \times f\times t}$ *τ* represents the temporal center of the window, and *f* is the frequency. The squared modulus of the STFT is referred to as the spectrogram [27].

(2) $${SPEC}_{x}\left(\tau ,f\right)={\left|{STFT}_{x}\left(\tau ,f\right)\right|}^{2}$$

The choice of window length is a critical aspect of time–frequency analysis because it determines the trade-off between temporal and frequency resolution. Shorter windows enable the detection of rapid changes in the signal, providing high temporal resolution but reducing the precision of frequency estimation [26]. In contrast, longer windows improve frequency resolution but average rapid variations in the signal, limiting the detection of transient events such as electromyographic bursts.

For EMG signals with highly nonstationary bursting activity, the window length also defines the time interval over which a pseudo-stationarity assumption can be made [10]. This condition is necessary for the local Fourier transform applied to each segment to provide valid spectral estimates, since the STFT assumes that the statistical properties of the signal remain constant within the temporal window.

In previous studies, the stationarity of BF EMG bursts in rats was evaluated using the Reverse Arrangement Test [10]. Mean squared power values were computed in non-overlapping 10 ms segments, a duration shorter than the minimum inter-spike interval of individual motor-unit action potentials (MUAPs), ensuring that each segment was not influenced by motor-unit firing rate. It was observed that, around the RMS peak, the EMG activity was stationary within a ~60 ms window across all conditions. In animals at weeks 5 and 6 post-lesion, EMG bursts remained stationary for longer periods, approximately 75 ms and up to ~230 ms, respectively. Thus, approximately 80% of BF EMG bursts satisfied stationarity criteria within ~60 ms around the peak [10]. Based on these findings, the spectrogram of each BF EMG signal was computed using an 80 ms Hamming window, a 75 ms overlap, and an FFT length of 160 points. This configuration provided a balance between temporal and frequency resolution, accurately capturing the rapid variations in the bursts without compromising the validity of the stationarity assumption.

From the resulting spectrograms, quantitative metrics representative of the spectral dynamics of the EMG bursts were extracted. In particular, mean frequency (F_mean_) and median frequency (F_median_) were computed throughout the contractile dynamics of the BF [9], enabling statistical comparisons across experimental conditions during the recruitment, sustained, and derecruitment phases of muscle contraction. Average F_mean_ and F_median_ values per contractile phase and per animal were obtained and used as the unit of analysis in group comparisons.

Because each experimental group (control and weeks 3–6 post-lesion) consisted of different animals, the design was considered between subjects. However, for statistical analysis, all contractions identified within each group were used as observation units, allowing analysis with a large number of samples per condition. Under this scheme, and given the size of the dataset, a one-way ANOVA was applied to evaluate differences between groups. When the ANOVA was significant, post hoc comparisons were performed using Tukey’s test to identify pairwise differences. This approach enabled a sensitive and consistent evaluation of group differences in metrics derived from the time–frequency analysis [9].

#### 2.3.2. Continuous Wavelet Transform

The continuous wavelet transform (CWT) is another widely used time–frequency representation for analyzing non-stationary signals. Unlike the spectrogram based on the STFT, which employs a fixed-duration window, the CWT simultaneously adapts temporal and frequency resolution through basis functions known as wavelets [26]. This provides a more flexible characterization of transient events and oscillations of different scales in the signal *x*(*t*). Mathematically, the CWT of a signal *x*(*t*) is defined as:

(3) $$W_{x}\left(\tau ,s\right)=\frac{1}{\sqrt{\left|s\right|}}\int_{-\infty }^{\infty }x\left(t\right)\psi^{*}\left(\frac{t-\tau }{s}\right)dt$$

where *ψ*(*t*) is the mother wavelet, *τ* denotes the temporal translation, *s* the scale factor, and ∗ the complex conjugate. The scale parameter s is inversely related to frequency: small scales allow the detection of high-frequency components with good temporal resolution, whereas large scales capture low-frequency components with greater frequency resolution.

The time–frequency map obtained from the CWT is expressed as wavelet energy (Equation (4)):

(4) $$P_{x}\left(\tau ,f\right)={\left|W_{x}\left(\tau ,s(f)\right)\right|}^{2}$$

where *s*(*f*) indicates the relationship between scale and effective frequency.

In this study, the Bump mother wavelet was used [28]. The choice of this wavelet is supported by its high spectral concentration and temporal compactness, properties that are particularly useful for electromyographic signals characterized by short, high-amplitude transient events such as motor-unit bursts. Unlike the Morlet wavelet, which yields a smoother representation of energy, the Bump wavelet enables a more precise detection of rapid changes in the spectral structure of the signal [29].

From the time–frequency maps obtained through the CWT, the same quantitative metrics used for the spectrograms, median frequency (F_median_) and mean frequency (F_mean_) were computed. For each animal, average F_mean_ and F_median_ values were calculated across the different contractile phases, constituting the unit of analysis for group comparisons. Statistical analysis was performed analogously to that used for the spectrograms, applying ANOVA and post hoc comparisons using Tukey’s test. This approach provides a consistent assessment of the spectral dynamics of EMG activity while ensuring comparability with the metrics obtained previously.

#### 2.3.3. Noise-Assisted Multivariate Empirical Mode Decomposition (NA-MEMD)

The EMG signals recorded during locomotion were also processed using the NA-MEMD algorithm, with the aim of obtaining an adaptive representation free from prior assumptions regarding the oscillatory content of the signals. This approach is particularly well suited for analyzing non-stationary and nonlinear signals, such as rapid muscle contractions, because it decomposes the signal into a set of intrinsic mode functions (IMFs), each associated with locally homogeneous oscillations in both time and frequency.

For this procedure, continuous EMG recordings (~2 min per animal during locomotion) were arranged into a multichannel matrix of dimensions N × T, where N corresponds to the number of animals and T to the number of temporal samples in each recording. Following the original scheme proposed by Rehman and Mandic [19], three channels of Gaussian white noise were added to the dataset, a characteristic requirement of NA-MEMD that helps stabilize the decomposition and reduce mode mixing [30].

A key characteristic of this analysis is that NA-MEMD was applied jointly to all recordings, performing a single decomposition on the multichannel matrix (including the noise channels). This approach ensures that the IMFs obtained for each animal correspond to the same global oscillatory modes, improving intersubject consistency and facilitating direct comparisons across animals and experimental conditions. No subject-specific independent decompositions were performed.

The NA-MEMD algorithm was implemented in MATLAB R2024a (The MathWorks, Inc., Natick, MA, USA) following the methodology described by Rehman and Mandic [30]. Once the IMFs were extracted, the Hilbert transform (HT) was applied to each component to estimate instantaneous frequency and instantaneous energy over time [22]. This strategy provides high temporal resolution for characterizing the frequency dynamics of each oscillatory mode without relying on global measures such as mean or median frequency. In this context, the HT representation offers a more precise description of the local spectral behavior of rapid contractions while preserving the adaptive and intrinsic nature of the decomposition.

#### 2.3.4. IMF Post-Processing

This subsection describes the post-processing procedures applied to the extracted IMFs, including burst segmentation and the computation of instantaneous frequency and energy-based features.

After decomposition, the EMG signals were segmented to identify biceps femoris contractions following the procedure previously described in [9]. This process allowed a precise delimitation of the intervals corresponding to each burst of muscle activity. Segmentation was applied synchronously to the IMF series so that each contraction was linked to its corresponding set of oscillatory components.

The HT was applied to each IMF to obtain instantaneous frequency and instantaneous energy time series. For each contraction phase of interest and for each IMF, two metrics were calculated: (1) an energy variable, defined as the mean instantaneous energy across the contraction phase, and (2) a frequency variable, defined as the mean instantaneous frequency over the same interval. These two variables allow each contraction to be represented in a two-dimensional space defined by frequency and energy, facilitating comparisons across experimental conditions and the identification of potential clustering patterns according to the dominant oscillatory mode.

#### 2.3.5. Statistical Analysis

The statistical analyses performed to evaluate group differences and the discriminative power of the extracted features are described below.

To evaluate the discriminative power of the features extracted from each IMF, a statistical approach combining multivariate and univariate analyses was employed. First, a multivariate analysis of variance (MANOVA) was applied, simultaneously considering mean instantaneous frequency and mean instantaneous energy to assess global differences between experimental groups. When MANOVA revealed significant effects, independent univariate ANOVAs were conducted for each variable in each contractile phase of the BF (recruitment, sustained, and derecruitment). Because multiple ANOVAs were performed, the resulting *p*-values were adjusted using the Holm–Bonferroni correction to control for Type I error associated with multiple comparisons. Only those ANOVAs that remained significant after correction were subjected to post hoc multiple comparisons. This procedure allowed identification of the specific IMFs showing differences between experimental conditions and, consequently, determination of which ones provided the greatest discriminative power in the context of neurodegenerative progression.

## 3. Results

**Figure 2:**
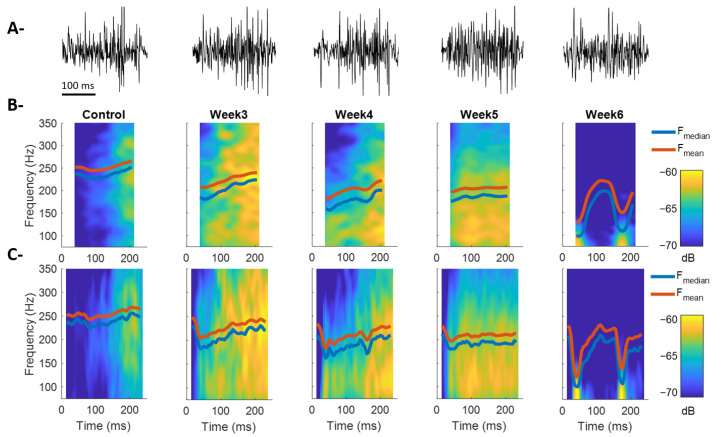
Time–frequency analysis based on spectrograms computed via the short-time Fourier transform (STFT). (**A**) EMG signals of the biceps femoris (BF) obtained from a single representative animal for each experimental condition (Control and Weeks 3, 4, 5, and 6 post-lesion). (**B**) Average spectrograms calculated from multiple contractions of the same animal in each experimental group (228, 154, 82, 232, and 216 EMG recordings for Control, Week 3, Week 4, Week 5, and Week 6, respectively). Temporal trajectories of mean frequency (F_mean_, orange) and median frequency (F_median_, blue) are superimposed on each spectrogram. Calculations were performed using an 80 ms Hamming window with 75 ms overlap. (**C**) Average spectrograms computed with a 30 ms window and 25 ms overlap, showing improved temporal resolution at the expense of lower frequency resolution.

Figure 2 shows the EMG recordings obtained during BF contractions in experimental animals under different post-lesion time conditions. The average duration of BF contractions was approximately 250 ms across all experimental conditions, determined by the speed of the testing platform [23]. Figure 2B displays the average spectrograms and the corresponding F_mean_ and F_median_ calculated from all recorded steps of a representative animal for each condition. In general terms, differences in the temporal evolution of the spectral components can be observed throughout the contraction, along with a decreasing trend in their average values as post-lesion time progresses. The regions without spectral information at the beginning and end of the contraction (~40 ms) result directly from the use of an 80 ms temporal window for spectrogram computation. To minimize this limitation, the analysis was repeated using a 30 ms window (Figure 2C), which reduced the loss of information to the first and last 15 ms of BF contractile activity. This adjustment allowed a more precise visualization of the spectral evolution during the initial phase of the contraction, albeit at the cost of lower frequency resolution. This inverse relationship between temporal and frequency resolution represents a critical consideration in the time–frequency analysis of bursting EMG signals and must be carefully evaluated when interpreting physiological implications.

**Figure 3:**
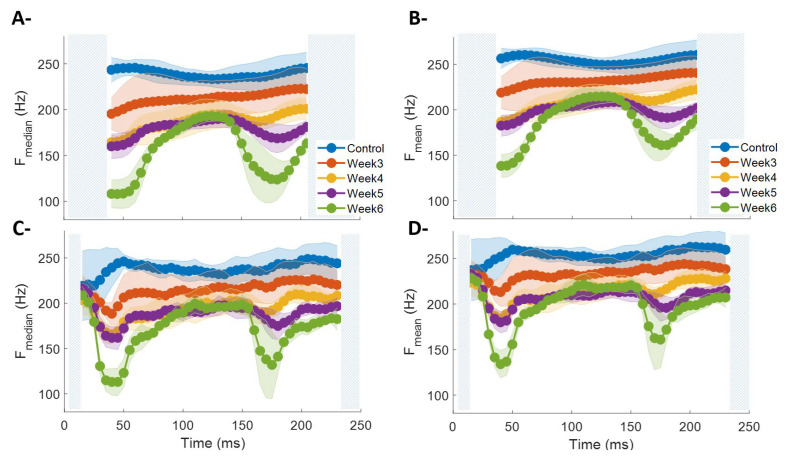
Temporal dynamics of the spectral components of the BF muscle during contraction across post-lesion weeks for all experimental animals in each group. (**A**) Median frequency (F_median_) throughout BF contraction in the control condition and Weeks 3–6 post-lesion. (**B**) Mean frequency (F_mean_) under the same experimental conditions. (**C**,**D**) Same as in A and B, respectively, but spectrograms were computed using a 30 ms temporal window instead of 80 ms. Hatched regions at the beginning and end of the curves (40 ms in (**A**,**B**) and 15 ms in (**C**,**D**)) indicate intervals affected by window edge effects in the spectrogram calculation. Lines represent group means, and shaded areas indicate the standard error.

Figure 3 shows the temporal evolution of F_median_ and F_mean_ during BF contraction for all experimental conditions and animals analyzed. In the control condition, both metrics exhibit higher and more stable values across the entire contraction, reflecting a spectral composition dominated by high-frequency components and sustained muscle activation (Figure 3A,B). In contrast, post-lesion conditions (Weeks 3–6) show a progressive reduction in F_mean_ and F_median_, accompanied by greater temporal variability during the contraction. In particular, Weeks 3, 4, and 5 display an increasing trend in F_median_ and F_mean_ over the course of the contraction, suggesting a modification in motor unit recruitment strategy, possibly compensatory in response to the loss of fast fibers [10]. The Week 6 condition is distinguished by a marked reduction in high-frequency components, especially at the beginning and end of the contraction, indicating a more severe degradation of the muscle’s spectral profile. This pattern appears to consolidate a tendency already emerging in Weeks 4 and 5, although to a more moderate extent (Figure 3A,B). Comparatively, spectrograms computed with the shorter 30 ms window (Figure 3C,D) allow improved temporal resolution of transient changes while maintaining the same overall trends.

Table 1 summarizes the multiple statistical comparisons performed using F_median_ as the spectral feature across the different experimental conditions. Spectrograms computed with both 80 ms and 30 ms windows revealed significant differences in most comparisons, highlighting the derecruitment phase as the most sensitive for detecting changes between conditions. In the initial contraction phase (recruitment), no significant differences were found between weeks 4 and 5. Similarly, during the sustained phase, no differences were identified between weeks 4, 5, and 6. Comparable results were obtained when using F_mean_ under the same experimental scenarios.

### 3.1. Time–Frequency Diagram via CWT

**Figure 4:**
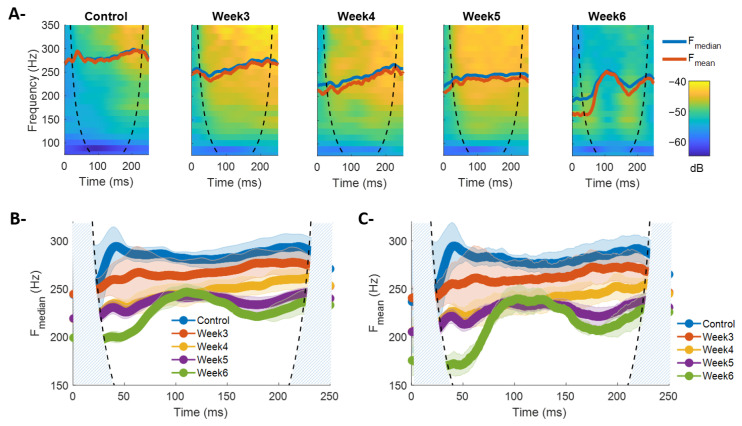
Time–frequency analysis of the biceps femoris (BF) muscle obtained via continuous wavelet transform (CWT) for different experimental conditions. (**A**) Average time–frequency maps from a representative animal for the control condition and Weeks 3–6 post-lesion. Over each map, the temporal trajectories of median frequency (F_median_, blue) and mean frequency (F_mean_, red) are shown. Dashed lines indicate the cones of influence delimiting the region of valid information in the wavelet analysis. (**B**,**C**) Average F_median_ (**B**) and F_mean_ (**C**) across BF contractions for all experimental animals in each group. Striped regions at the beginning and end of the curves represent intervals affected by the validity limits of the wavelet analysis. Lines indicate group mean values, and shaded areas represent the standard error of the mean.

Figure 4 shows the time–frequency maps obtained via CWT for BF in control animals and those at Weeks 3–6 post-lesion. Each map represents the average of all contractions recorded in a single animal for a given experimental condition. Superimposed on these maps are the temporal trajectories of F_mean_ and F_median_ along with the cones of influence delimiting valid CWT regions. Overall, the temporal trends observed in both spectral metrics are consistent with those derived from STFT-based spectrograms (Figure 3), showing a progressive reduction in high-frequency components and more pronounced temporal modulation in post-lesion conditions. However, the CWT provides substantial analytical advantages by not requiring an explicitly defined fixed temporal window. Its intrinsic multiscale nature adaptively adjusts temporal and frequency resolution, thereby avoiding the trade-offs inherent to STFT window selection and enabling a more precise characterization of transient events and spectral evolution in EMG signals.

Figure 4B,C show the average F_mean_ and F_median_ time series obtained for all animals within each experimental group. Compared with the metrics derived from spectrograms, the absolute values obtained via CWT are slightly higher, which may be attributed to the method’s greater sensitivity to high-frequency transient components captured by the Bump wavelets, especially during the recruitment phase. It is also observed that information corresponding to the initial phase of the contraction is partially constrained when longer-duration wavelets are used. This effect is expected since greater wavelet duration improves frequency resolution at the expense of temporal resolution. In this context, CWT achieves superior frequency resolution, evidenced by lower dispersion (standard deviation) in the F_mean_ and F_median_ measurements, particularly in conditions with reduced spectral content (Weeks 4–6).

Overall, the application of CWT provides a representation complementary to that of the spectrogram. While STFT allows a direct comparison with classical methods via uniform temporal resolution, CWT offers a more detailed view of the hierarchical organization of EMG spectral components, capturing with higher precision the dynamic changes associated with contraction phases and post-lesion progression.

The spectral dynamics metrics, both those derived from STFT-based spectrograms and those obtained from CWT time–frequency diagrams, reveal distinct post-lesion trends across the different phases of BF contraction. During the recruitment phase, a partial reduction in frequency is observed with increasing post-lesion time, except between Weeks 4 and 5, which exhibit similar dynamics. During the sustained phase (80–150 ms), frequency shows a decreasing trend in control, Week 3, and the subgroup composed of Weeks 4–6, which share comparable dynamics. Finally, during derecruitment, all evaluated conditions display a progressive reduction in frequency as a function of post-lesion time.

CWT-based time–frequency diagrams revealed patterns highly consistent with those derived from spectrograms. Table 2 summarizes the multiple comparisons performed using F_median_ and F_mean_. In general, significant differences were observed in most experimental scenarios and across all three contraction phases, again highlighting derecruitment as the most sensitive phase for discriminating between conditions. During recruitment, comparisons between Weeks 4 and 5 showed no significant differences, whereas during the sustained phase no differences were detected between Weeks 4, 5, and 6. These results mirror the patterns observed with STFT, confirming that both F_median_ and F_mean_ exhibit strong agreement across time–frequency methods for characterizing spectral changes associated with the experimental conditions.

### 3.2. Time–Frequency Analysis via NA-MEMD

**Figure 5:**
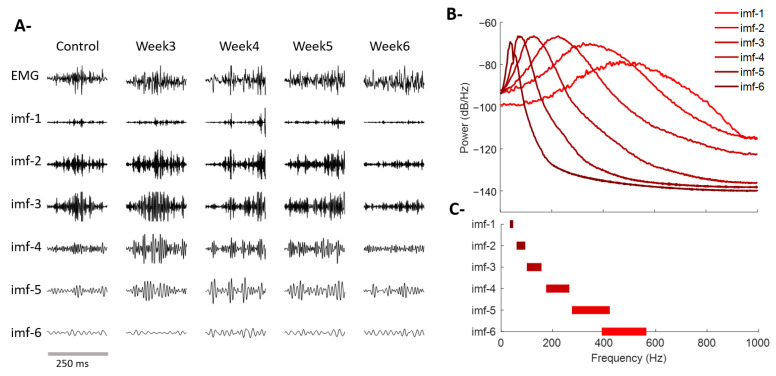
Multivariate empirical decomposition of EMG via NA-MEMD and spectral characterization of intrinsic mode functions (IMFs). (**A**) Representative EMG signals recorded during rapid contractions of the biceps femoris under control conditions and at Weeks 3, 4, 5, and 6 post-lesion. Below, the six intrinsic mode functions (IMF-1 to IMF-6) resulting from NA-MEMD decomposition are shown. (**B**) Average power spectra for each IMF. (**C**) Predominant frequency range associated with each mode. The staggered, non-overlapping distribution confirms that each IMF occupies a well-defined frequency interval.

Figure 5 shows a representative example of EMG decomposition via NA-MEMD and the spectral characterization of the resulting modes. Figure 5A depicts the original signal and its intrinsic mode functions (IMFs 1 through 6) for a control recording and for each post-lesion week (Weeks 3–6). The method successfully separated the signal into well-defined oscillatory components, each associated with a specific frequency range. Higher-order modes (IMF-1 to IMF-3) captured fast, transient oscillations of the contraction, whereas intermediate and slower modes (IMF-4 to IMF-6) reflected lower-frequency patterns with greater temporal stability. Figure 5B presents the average power spectra of each IMF, which exhibit a hierarchical frequency organization with minimal overlap between modes. This distribution confirms that the decomposition achieved an effective separation of the oscillatory components present in the muscle signal. Finally, Figure 5C summarizes the predominant frequency ranges of each mode, showing well-defined and non-overlapping spectral bands, supporting the suitability of NA-MEMD for representing EMG signals in terms of adaptive oscillatory components.

**Figure 6:**
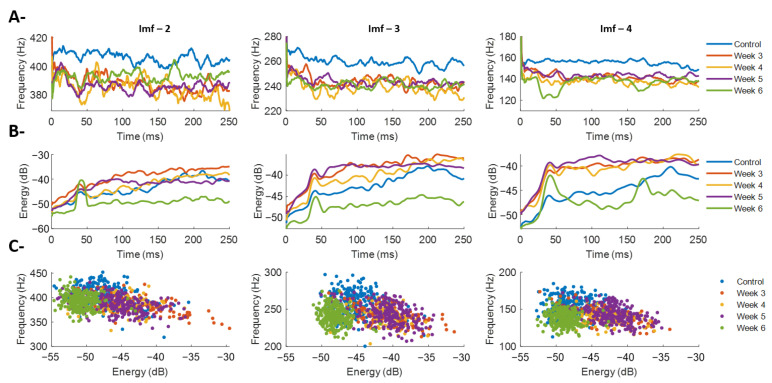
Instantaneous frequency and energy analysis of the biceps femoris (BF) obtained via the Hilbert transform applied to intrinsic mode functions (IMFs 2–4) for the different experimental conditions. (**A**) Average instantaneous frequency throughout BF contraction. Each panel corresponds to a different mode function: left panel shows IMF-2, center panel IMF-3, and right panel IMF-4. Within each panel, the temporal trajectories of instantaneous frequency for all experimental conditions (control and Weeks 3–6 post-lesion) are displayed, allowing comparison of the frequency dynamics specific to each IMF. (**B**) Average instantaneous energy obtained for each IMF. As in (**A**), each panel corresponds to a particular mode function (IMF-2 on the left, IMF-3 in the center, IMF-4 on the right). The curves show the temporal evolution of instantaneous energy for each experimental condition, highlighting differences in early transients and stability during the sustained contraction phase. (**C**) Frequency–energy relationship for each individual BF contraction. Each panel presents the average instantaneous frequency and energy values corresponding to a specific IMF (IMF-2, IMF-3, and IMF-4, from left to right). Each point represents a single contraction of an animal under a given experimental condition (control or Weeks 3–6 post-lesion), illustrating the joint distribution of both metrics for each mode.

Figure 6A presents the average instantaneous frequency obtained from the Hilbert transform applied to IMFs 2 through 4 for each post-lesion condition. Overall, no clearly progressive trend is observed, unlike the patterns identified in F_mean_ and F_median_ derived from STFT and CWT. IMF-2 shows that the control condition exhibits the highest frequencies, whereas post-lesion conditions remain within a narrow range of approximately 380–400 Hz (Figure 6A, left). IMFs 3 and 4 show similar patterns at lower frequencies. Notably, IMF-4 reveals a decrease in frequency content during the recruitment phase for Week 6, consistent with the reductions observed in F_mean_ and F_median_ reported earlier. This finding highlights an inherent limitation of mode-based decomposition: the technique does not reflect a progressively decreasing frequency pattern over post-lesion time. This limitation may arise because frequency variations distribute across modes, so each IMF provides only a partial and mode-specific representation of the spectral dynamics associated with each post-lesion stage.

Figure 6B shows the average instantaneous energy obtained via the Hilbert transform for each experimental condition across IMFs 2–4. Overall, the temporal dynamics of energy do not exhibit a systematic trend with post-lesion time, regardless of the mode considered. Nevertheless, some specific patterns can be identified. For IMF-2, a marked increase in energy is observed during recruitment, followed by stabilization after ~100 ms (Figure 6B, left). This behavior contrasts with the relatively stable instantaneous frequency for the same mode (Figure 6A, left), suggesting that the mode decomposition may capture complementary time–frequency characteristics through both instantaneous energy and frequency. Additionally, positive energy transients appear during the initial recruitment phase, particularly around ~50 ms, and are more pronounced in Week 6 animals (Figure 6B). This feature may reflect abrupt or synchronized activation of motor unit groups contributing to these frequency scales, consistent with impaired early recruitment associated with progressive dopaminergic degeneration.

Figure 6C illustrates the relationship between average instantaneous frequency and average instantaneous energy for each contraction, where each point represents a single BF contraction in each experimental condition. Across IMFs 2–4, a consistent pattern emerges: contractions with higher energy tend to exhibit lower frequencies, whereas those with lower energy present higher frequencies. This behavior appears across all experimental groups. Additionally, the dispersion of points within each condition reveals differences in inter-contraction variability. The control group displays a broader range of frequencies and energies, whereas post-lesion conditions exhibit a more concentrated distribution around reduced values of both parameters. This progressive narrowing of the frequency–energy space suggests that contractions become more homogeneous as post-lesion time advances. Collectively, the frequency–energy representation complements the observations from Figure 6A,B by providing insight into the average behavior of each contraction and its organization within each experimental condition.

The MANOVA, which jointly assessed average instantaneous frequency and energy for each IMF across the three contraction phases, revealed significant differences between experimental conditions. This result indicates that the effects of neurodegenerative progression are reflected simultaneously in the dynamic features of the mode functions, justifying subsequent pairwise group comparisons.

Table 3 and Table 4 present the *p*-values from the multiple comparisons adjusted using Bonferroni correction, performed on average instantaneous frequency and energy, respectively. The results show that changes associated with dopaminergic lesion can be differentiated through the oscillatory properties of IMFs 2, 3, and 4, even when some trends are not fully apparent from visual inspection of individual curves.

Significant differences were observed in most contrasts between the control group and post-lesion Weeks 3–6, particularly during the sustained and derecruitment phases, suggesting that these stages of the contractile cycle are especially sensitive to the effects of neurodegeneration. Higher-order IMFs (IMF-3 and IMF-4) also demonstrated greater discriminative capacity, reflecting robust changes in both instantaneous frequency and energy. Additionally, several contrasts between consecutive weeks were significant, indicating that lesion effects evolve progressively and non-uniformly over time.

Overall, these findings confirm that the combination of instantaneous frequency and instantaneous energy derived from empirical mode decomposition constitutes a sensitive marker of alterations in EMG time–frequency dynamics, providing complementary evidence on how muscle activity reorganizes during neurodegenerative progression in a PD model.

## 4. Discussion

### 4.1. Spectrogram and CWT Analyses

In this study, we characterized the temporal dynamics of the spectral content of EMG bursts generated by BF contractions in animals at different post-lesion time points in a PD model. To this end, we employed spectrograms based on STFT, CWT, and the nonlinear empirical decomposition NA-MEMD. This approach provides a complementary perspective to our previous work, in which we analyzed the spectral and morphological features of locomotion-related EMG bursts using classical frequency-domain parameters and stationarity assessments [9,10].

Analysis of the frequency metrics derived from STFT- and CWT-based representations revealed phase-dependent trends across the different stages of muscle contraction. During the recruitment phase, a partial reduction in frequency was observed with increasing post-lesion time, except in Weeks 4 and 5, which displayed dynamics similar to the control condition. In the sustained contraction phase, a marked drop in frequency was evident in the control, Week 3, and Week 6 conditions, whereas Weeks 4 and 5 maintained comparable frequency dynamics between 80 and 150 ms. Finally, during derecruitment, all post-lesion conditions showed a progressive reduction in high-frequency components as post-lesion time increased. Taken together, these findings suggest that progressive neurodegeneration due to nigrostriatal dopaminergic loss affects the spectral composition of muscle activity in a phase-dependent manner, indicating that neuromuscular control mechanisms are altered in a temporally specific fashion.

Physiologically, the rat biceps femoris is composed primarily of fast-fatigable fibers (49%) and fast fatigue-resistant fibers (47%), with a smaller proportion (≈4%) of slow motor units [31]. Under normal conditions, the recruitment of fast motor units ensures rapid and powerful contractions required during locomotion [12]. However, in 6-OHDA-lesioned animals, the spectral shift toward lower frequencies—consistently observed both here and in previous studies—may reflect structural remodeling of the muscle as well as compensatory functional adaptations in motor unit recruitment [32,33,34,35].

Consistent with this interpretation, multiple studies have demonstrated that dopaminergic depletion in the substantia nigra pars compacta leads to alterations in basal ganglia–thalamocortical circuitry that secondarily influence spinal motoneuron output [36,37]. Such central alterations can disrupt the normal pattern of motor unit activation, producing irregular, low-threshold, and intermittent discharges, as reported in both PD patients and animal models [34,35]. These irregularities may manifest as phase-dependent spectral modulations, as identified in this work, in which the early phase of the contraction becomes progressively less stable while later phases exhibit a compressed spectral content.

Previous stationarity analyses of EMG signals [10] showed that, as post-lesion time increased, both variance and autocovariance-based stationarity tended to stabilize. This stabilization likely reflects the predominance of slow, low-threshold fibers and the loss of fast, phasic components, consistent with a transition from fast to slow muscle phenotypes, as previously described by Nakamura et al. [38]. The time–frequency findings presented here reinforce this interpretation: the reduction in high-frequency content during recruitment and derecruitment parallels the progressive loss of dynamic motor-unit firing modulation, whereas the intermediate contraction phase appears to exhibit partial compensatory recruitment capable of maintaining transient frequency stability (Weeks 4–5).

This temporal evolution may be associated with denervation and reinnervation processes occurring as a consequence of nigrostriatal dopaminergic loss. Remodeling processes may lead to increased motor-unit clustering and the synchronization of action potentials [38,39,40], both of which could contribute to the narrowing of the EMG power spectrum and a reduction in spectral variability. Consequently, the observed compression of EMG frequency content across post-lesion time may be interpreted as the electrophysiological signature of a muscle increasingly dominated by slower and more synchronized motor units, with reduced flexibility to respond to contractile demands.

The time–frequency framework proposed in this work provides additional insight into these neuromuscular adaptations by revealing not only static spectral changes but also how such changes evolve dynamically throughout the contractile cycle. This phase-resolved analysis highlights that different subgroups of motor units—possibly with distinct conduction velocities and recruitment thresholds—participate sequentially during the onset, maintenance, and termination of muscle activation. The altered patterns observed in lesioned animals indicate that the normal temporal coordination among these subgroups becomes progressively compromised, leading to less efficient recruitment and slower deactivation dynamics.

Overall, combining previous findings with the present time–frequency approach supports a unified interpretation: dopaminergic degeneration induces a transition toward more stable but less flexible EMG activity, reflected in bandwidth narrowing, increased amplitude stationarity, and phase-dependent loss of high-frequency components. These results strengthen the hypothesis that spectral compression and altered recruitment dynamics may serve as functional biomarkers of neuromuscular degeneration in animal models of PD.

### 4.2. NA-MEMD

Beyond the phase-dependent trends identified using STFT and CWT, NA-MEMD, combined with instantaneous frequency and energy analysis derived from the HT, enabled the evaluation of EMG dynamics from a complementary perspective based on intrinsic oscillators. Unlike linear time–frequency methods, which operate on global spectral representations, EMD separates the signal into adaptive bands that capture local oscillatory patterns potentially associated with subgroups of motor units with similar physiological characteristics.

However, the results showed that instantaneous frequencies of modes 2–4 did not exhibit the consistent progression toward lower values with increasing post-lesion time that was observed in F_mean_ and F_median_ metrics derived from STFT and CWT. This lack of a progressive trend can be explained by the nature of empirical decomposition: the spectral changes associated with dopaminergic lesion are not concentrated exclusively within a single characteristic band but rather distributed across a set of frequency scales that EMD assigns to consecutive modes. As a consequence, each IMF captures only a fraction of the lesion-induced spectral shift, thereby diluting the ability of any single mode to represent the full temporal evolution of frequency content.

Nevertheless, certain characteristics of the IMFs were consistent with findings from STFT/CWT. For example, mode 4 displayed a clear frequency drop during the recruitment phase in animals at six weeks post-lesion, in agreement with the loss of fast components previously identified in classical time–frequency representations. This suggests that, although spectral redistribution is spread across multiple modes, the most pronounced changes associated with the loss of high-frequency components may emerge in specific modes containing oscillations most closely associated with fast motor units.

Regarding instantaneous energy, the analysis revealed that none of the IMFs exhibited systematic evolution with post-lesion time, but transient, phase-specific dynamics were observed. A particularly relevant finding was the abrupt energy increase around 50 ms in later post-lesion weeks, especially Week 6. This feature suggests the possible presence of brief, synchronized activations within high-threshold motor-unit subpopulations or the emergence of a more abrupt initial recruitment associated with neuromuscular remodeling [10]. Although this observation does not allow definitive interpretation, it indicates that instantaneous energy may capture transient desynchronization–resynchronization signatures not readily apparent in STFT/CWT representations.

In this context, the joint frequency–energy representation (Figure 6C) highlights that contractions from the control group and from later post-lesion stages, particularly Weeks 5 and 6, become more clearly discriminated. This separation suggests that advanced neurodegeneration is associated not with a gradual linear shift in individual parameters, but with a qualitative reorganization of activation dynamics that emerges in the combined frequency–energy domain. Overall, the results derived from empirical decomposition emphasize that the phase-dependent spectral modulation observed in linear time–frequency methods does not originate from a single oscillatory generator but rather emerges from the interaction of multiple intrinsic scales. This distributed nature of frequency reorganization explains why IMFs do not individually exhibit the clear temporal progression observed with STFT and CWT. Nevertheless, when instantaneous frequency and energy are analyzed jointly, EMD provides additional information on local dynamics that may reflect compensatory mechanisms, such as transient increases in synchronization or the premature activation of fast-conducting motor units, which are not fully captured in global spectrograms.

Thus, combining empirical decomposition with linear time–frequency methods strengthens the study’s overall conclusion: dopaminergic lesion progression alters not only the global distribution of spectral content but also affects intrinsic oscillatory components in a heterogeneous, scale-specific manner. This multimethod framework delineates a more precise map of how recruitment mechanisms, synchrony, and dynamic modulation reorganize across post-lesion time, providing further evidence that muscle activity in a neurotoxic Parkinson’s model becomes more stable, less flexible, and supported by a progressively narrower set of oscillatory scales.

### 4.3. Methodological Considerations of the Analytical Approaches

This study employed three complementary time–frequency approaches, STFT-based spectrograms, CWT, and NA-MEMD combined with Hilbert analysis, to characterize changes in the spectral dynamics of muscle contractions in an animal model of PD. Each method introduces assumptions, limitations, and advantages that shape the interpretation of derived metrics and therefore must be considered when comparing their outcomes.

First, spectrogram analysis relies on fixed-length time windows, which entails an inherent trade-off between temporal and frequency resolution. For the characterization of myoelectric signals with rapid temporal variations, this trade-off may limit the ability to detect transient spectral fluctuations, particularly when the window required to achieve adequate frequency resolution exceeds the effective duration of the contraction. Nonetheless, the regular structure and low computational cost make STFT a robust method for obtaining global parameters such as F_mean_ and F_median_, provided that window selection and boundary corrections are applied consistently.

Second, CWT offers scale-dependent adaptive resolution, allowing an improved characterization of transient events by increasing temporal resolution for high-frequency components. This property is advantageous when analyzing EMG signals whose spectral content reorganizes dynamically throughout contraction. However, CWT introduces dependencies on the choice of wavelet and its temporal width, which may influence metric estimation. Moreover, redundancy of the time–scale map and the absence of a strict frequency-space partition complicate direct comparison with methods based on conventionally defined frequency bands. Even so, spectral metrics derived from CWT, such as continuous-scale F_mean_ and F_median_, remain useful for describing global trends in non-stationary signals.

Finally, NA-MEMD yields IMFs that adapt to the signal without assuming a fixed spectral basis. This property provides a significant advantage for analyzing nonlinear and non-stationary signals, as each IMF represents a physically meaningful oscillation inherent to the data. Subsequent application of the Hilbert Transform to each IMF yields instantaneous frequency and energy time series with maximal temporal resolution, enabling a more specific characterization of contractile dynamics than traditional approaches. However, this method also has limitations. First, the decomposition may be affected by phenomena such as mode mixing, although the inclusion of auxiliary noise and the multivariate nature of NA-MEMD partially mitigate this issue. Second, IMFs do not represent standard frequency bands but rather emergent components of the signal, meaning that comparisons across subjects or conditions require consistent mode-selection criteria. Finally, sensitivity to the amount of added noise and to the number of channels used in the decomposition represents a critical aspect for reproducibility.

Overall, combining these three methods provides a complementary view of the spectral organization of muscle contractions: whereas STFT and CWT yield global spectral metrics within well-established analytical frameworks, NA-MEMD + Hilbert analysis offers a finer description of instantaneous dynamics. Integrating these approaches—while acknowledging both their strengths and limitations—is essential for appropriately interpreting the patterns observed across experimental conditions in this animal model.

Importantly, this complementary perspective is not only relevant for methodological rigor within experimental research but may also have implications beyond the present animal model. Understanding how different analytical frameworks capture distinct aspects of EMG dynamics is essential when considering their potential extension to applied or clinical contexts, where signal quality, recording conditions, and interpretative constraints may differ substantially. In this sense, the methodological considerations discussed above provide a necessary foundation for evaluating the translational relevance of advanced time–frequency EMG analysis.

### 4.4. Clinical Implications and Translational Perspectives

Although the present study was conducted in a preclinical Parkinsonian model using controlled experimental conditions and subcutaneous electrodes to optimize signal quality, the methodological insights derived from this work may have broader translational relevance. The identification of phase-dependent spectral compression and reduced dynamic flexibility across post-lesion time suggests that alterations in the temporal organization of motor-unit recruitment may serve as functional electrophysiological markers of neuromuscular reorganization.

In clinical settings, EMG recordings are commonly obtained using surface electrodes adhered to the skin, which introduce additional challenges such as lower signal-to-noise ratio, cross-talk between adjacent muscles, and motion artifacts. Under these conditions, robust preprocessing strategies remain essential to ensure the reliable interpretation of spectral metrics [9]. Time–frequency approaches capable of characterizing transient and highly nonstationary bursts may offer advantages over purely stationary or global spectral analyses, particularly when muscle activation occurs in short, irregular epochs.

The multiscale framework explored in this study may enhance the detection of subtle alterations in motor control, especially in early or progressive stages of movement disorders. These findings are conceptually aligned with our previous work based on stationarity testing and permutation entropy, in which progressive restructuring of EMG temporal organization and reductions in signal complexity were identified as potential biomarkers of Parkinsonian neuromuscular remodeling [9,10]. Together, these analytical strategies suggest that combining stationarity metrics, complexity measures, and time–frequency characterization may provide a more comprehensive assessment of contractile dynamics than any single framework alone.

Beyond Parkinsonian models, this integrative analytical approach may also be extended to the evaluation of muscle fatigue, rehabilitation monitoring, and motor retraining processes, where changes in recruitment efficiency, synchronization patterns, and spectral distribution are expected [41,42]. By enabling a phase-resolved and scale-specific characterization of EMG activity, advanced signal-processing tools may contribute to the development of more sensitive electrophysiological markers for tracking disease progression or therapeutic response in both experimental and clinical neurorehabilitation contexts.

## 5. Conclusions

This study provides a structured comparative evaluation of three time–frequency methodologies—STFT-based spectrogram, CWT, and NA-MEMD coupled with Hilbert transform analysis—for the characterization of highly nonstationary EMG bursts in a Parkinsonian animal model. The results demonstrate that these approaches do not offer redundant information but rather highlight complementary dimensions of neuromuscular reorganization. Linear time–frequency methods (STFT and CWT) were particularly effective in capturing global and phase-dependent spectral reorganization patterns associated with progressive nigrostriatal degeneration, offering stable and interpretable metrics such as F_mean_ and F_median_. In contrast, NA-MEMD combined with instantaneous frequency and energy analysis enabled the identification of local, scale-specific, and transient dynamics that remained partially concealed in conventional spectrogram-based representations. Together, these findings indicate that dopaminergic degeneration alters EMG frequency modulation in a phase-dependent and multiscale manner. The main methodological contribution of this work lies in establishing explicit criteria for selecting and integrating complementary time–frequency tools according to the analytical objective, thereby advancing the rigorous characterization of nonstationary muscle activity. This comparative framework may support the development of more sensitive electrophysiological markers of neuromuscular reorganization in both experimental and translational contexts.

## Figures and Tables

**Table 1:** Significance values (*p*-values) obtained from statistical analysis and multiple comparisons for F_median_. F_median_ for each contraction phase was extracted from spectrograms computed using 80 ms and 30 ms windows.

	Spectrogram (Window Length = 80 ms)			Spectrogram (Window Length = 30 ms)		
	Recruitment Phase	Steady-State Phase	Derecruitment Phase	Recruitment Phase	Steady-State Phase	Derecruitment Phase
control vs. week 3	*p* < 0.001	*p* < 0.001	*p* < 0.001	*p* < 0.001	*p* < 0.001	*p* < 0.001
control vs. week 4	*p* < 0.001	*p* < 0.001	*p* < 0.001	*p* < 0.001	*p* < 0.001	*p* < 0.001
control vs. week 5	*p* < 0.001	*p* < 0.001	*p* < 0.001	*p* < 0.001	*p* < 0.001	*p* < 0.001
control vs. week 6	*p* < 0.001	*p* < 0.001	*p* < 0.001	*p* < 0.001	*p* < 0.001	*p* < 0.001
week 3 vs. week 4	*p* < 0.001	*p* < 0.001	*p* < 0.001	*p* < 0.001	*p* < 0.001	*p* < 0.001
week 3 vs. week 5	*p* < 0.001	*p* < 0.001	*p* < 0.001	*p* < 0.001	*p* < 0.001	*p* < 0.001
week 3 vs. week 6	*p* < 0.001	*p* < 0.001	*p* < 0.001	*p* < 0.001	*p* < 0.001	*p* < 0.001
week 4 vs. week 5	0.990	1.000	*p* < 0.001	0.382	0.996	*p* < 0.001
week 4 vs. week 6	*p* < 0.001	0.162	*p* < 0.001	*p* < 0.001	0.766	*p* < 0.001
week 5 vs. week 6	*p* < 0.001	0.133	*p* < 0.001	*p* < 0.001	0.926	*p* < 0.001

**Table 2:** Significance values (*p*-values) obtained from statistical analysis and multiple comparisons for F_median_ and F_mean_. Both metrics were extracted from time–frequency diagrams estimated via CWT for each contraction phase.

	CWT (F_median_)			CWT (F_mean_)		
	Recruitment Phase	Steady-State Phase	Derecruitment Phase	Recruitment Phase	Steady-State Phase	Derecruitment Phase
control vs. week 3	*p* < 0.001	*p* < 0.001	*p* < 0.001	*p* < 0.001	*p* < 0.001	*p* < 0.001
control vs. week 4	*p* < 0.001	*p* < 0.001	*p* < 0.001	*p* < 0.001	*p* < 0.001	*p* < 0.001
control vs. week 5	*p* < 0.001	*p* < 0.001	*p* < 0.001	*p* < 0.001	*p* < 0.001	*p* < 0.001
control vs. week 6	*p* < 0.001	*p* < 0.001	*p* < 0.001	*p* < 0.001	*p* < 0.001	*p* < 0.001
week 3 vs. week 4	*p* < 0.001	*p* < 0.001	*p* < 0.001	*p* < 0.001	*p* < 0.001	*p* < 0.001
week 3 vs. week 5	*p* < 0.001	*p* < 0.001	*p* < 0.001	*p* < 0.001	*p* < 0.001	*p* < 0.001
week 3 vs. week 6	*p* < 0.001	*p* < 0.001	*p* < 0.001	*p* < 0.001	*p* < 0.001	*p* < 0.001
week 4 vs. week 5	0.989	0.832	*p* < 0.001	0.998	1.000	*p* < 0.001
week 4 vs. week 6	*p* < 0.001	0.941	*p* < 0.001	*p* < 0.001	0.416	*p* < 0.001
week 5 vs. week 6	*p* < 0.001	1.000	*p* < 0.001	*p* < 0.001	0.358	*p* < 0.001

**Table 3:** *p*-values obtained from pairwise statistical comparisons between the different experimental conditions (Control and Weeks 3–6 post-lesion) for instantaneous frequency estimated from the Hilbert transform of each intrinsic mode function (IMF-2, IMF-3, and IMF-4). For each IMF, values were calculated as the average instantaneous frequency within each contraction phase of the EMG burst: recruitment phase (RP), sustained phase (SP), and derecruitment phase (DP). *p*-values are shown for all experimental pairwise combinations.

	IMF-2			IMF-3			IMF-4		
	RP	SS	DP	RP	SS	DP	RP	SS	DP
control vs. week 3	*p* < 0.001	*p* < 0.001	*p* < 0.001	*p* < 0.001	*p* < 0.001	*p* < 0.001	*p* < 0.001	*p* < 0.001	*p* < 0.001
control vs. week 4	*p* < 0.001	*p* < 0.001	*p* < 0.001	*p* < 0.001	*p* < 0.001	*p* < 0.001	*p* < 0.001	*p* < 0.001	*p* < 0.001
control vs. week 5	*p* < 0.001	*p* < 0.001	*p* < 0.001	*p* < 0.001	*p* < 0.001	*p* < 0.001	*p* < 0.001	*p* < 0.001	*p* < 0.001
control vs. week 6	*p* < 0.001	*p* < 0.001	*p* < 0.001	*p* < 0.001	*p* < 0.001	*p* < 0.001	*p* < 0.001	*p* < 0.001	*p* < 0.001
week 3 vs. week 4	0.341	1.000	0.998	0.365	0.340	0.999	0.143	0.511	0.200
week 3 vs. week 5	0.161	0.589	0.886	0.481	0.686	0.987	0.993	0.869	0.607
week 3 vs. week 6	0.973	0.094	*p* < 0.001	0.984	0.370	1.000	*p* < 0.001	0.811	0.001
week 4 vs. week 5	1.000	0.696	0.994	0.010	0.890	0.966	0.040	0.099	0.005
week 4 vs. week 6	0.549	0.330	0.024	0.536	0.980	1.000	0.340	0.920	0.930
week 5 vs. week 6	0.307	*p* < 0.001	0.003	0.095	0.986	0.961	*p* < 0.001	0.128	*p* < 0.001

**Table 4:** *p*-values obtained from pairwise statistical comparisons between the different experimental conditions (Control and Weeks 3–6 post-lesion) for instantaneous energy estimated from the Hilbert transform of each intrinsic mode function (IMF-2, IMF-3, and IMF-4). For each IMF, values were calculated as the average instantaneous energy within each contraction phase of the EMG burst: recruitment phase (RP), sustained phase (SP), and derecruitment phase (DP). *p*-values are shown for all experimental pairwise combinations.

	IMF-2			IMF-3			IMF-4		
	RP	SS	DP	RP	SS	DP	RP	SS	DP
control vs. week 3	0.063	*p* < 0.001	*p* < 0.001	*p* < 0.001	*p* < 0.001	*p* < 0.001	*p* < 0.001	*p* < 0.001	*p* < 0.001
control vs. week 4	0.999	0.939	1.000	0.033	0.239	0.343	*p* < 0.001	*p* < 0.001	*p* < 0.001
control vs. week 5	0.039	0.077	0.538	*p* < 0.001	*p* < 0.001	0.005	*p* < 0.001	*p* < 0.001	*p* < 0.001
control vs. week 6	1.000	0.667	*p* < 0.001	0.089	0.063	*p* < 0.001	0.687	0.447	0.212
week 3 vs. week 4	0.372	*p* < 0.001	0.005	0.036	0.001	0.049	1.000	0.677	0.972
week 3 vs. week 5	1.000	*p* < 0.001	*p* < 0.001	*p* < 0.001	0.937	0.042	0.008	0.001	1.000
week 3 vs. week 6	0.047	*p* < 0.001	*p* < 0.001	*p* < 0.001	*p* < 0.001	*p* < 0.001	*p* < 0.001	*p* < 0.001	*p* < 0.001
week 4 vs. week 5	0.363	0.813	0.827	*p* < 0.001	0.003	0.966	0.041	*p* < 0.001	0.943
week 4 vs. week 6	0.999	0.407	0.016	*p* < 0.001	0.001	*p* < 0.001	*p* < 0.001	*p* < 0.001	*p* < 0.001
week 5 vs. week 6	0.026	0.001	0.034	*p* < 0.001	*p* < 0.001	*p* < 0.001	*p* < 0.001	*p* < 0.001	*p* < 0.001

## Data Availability

The experimental data analyzed in this study are publicly available in the dataset described in: [23]. The dataset can be accessed at https://doi.org/10.1016/j.dib.2021.107712.
